# Echocardiography can accurately estimate pulmonary artery wedge pressure without left atrial volume information—diagnostic and prognostic performance

**DOI:** 10.1093/ehjimp/qyaf082

**Published:** 2025-06-13

**Authors:** Thomas Lindow, Aristomenis Manouras, Geoff Strange, Per Lindqvist, David Playford, Odd Bech-Hanssen, Martin Ugander

**Affiliations:** Department of Pulmonary Medicine, Allergology, and Palliative Medicine, Clinical Sciences, Lund University, Box 117, 221 00 Lund, Sweden; Department of Clinical Physiology, Department of Research and Development, Region Kronoberg, Växjö Central Hospital, 351 88 Växjö, Sweden; Kolling Institute, Royal North Shore Hospital, and University of Sydney, St Leonards, Sydney, New South Wales 2065, Australia; Heart and Vascular Center, Unit of Heart Failure, Arrhythmia and GUCH, Karolinska University Hospital, Stockholm, Sweden; Department of Medicine, Karolinska Institutet, Solna, Sweden; School of Medicine, University of Notre Dame, Fremantle, Western Australia, Australia; Faculty of Medicine and Health, University of Sydney, Sydney, New South Wales, Australia; Department of Cardiology, Royal Prince Alfred Hospital, Sydney, New South Wales, Australia; Heart Research Institute, Sydney, New South Wales, Australia; Departments of Diagnostics and Intervention, Clinical Physiology, Umeå University, Umeå, Sweden; School of Medicine, University of Notre Dame, Fremantle, Western Australia, Australia; Department of Clinical Physiology, Sahlgrenska University Hospital, Gothenburg, Sweden; Institute of Medicine, Sahlgrenska Academy at the University of Gothenburg, Gothenburg, Sweden; Kolling Institute, Royal North Shore Hospital, and University of Sydney, St Leonards, Sydney, New South Wales 2065, Australia; Department of Clinical Physiology, Karolinska University Hospital, and Karolinska Institutet, Stockholm, Sweden

**Keywords:** left ventricular filling pressures, heart failure, echocardiography, left atrium, Doppler

## Abstract

**Aims:**

A quantitative estimate of pulmonary artery wedge pressure (PAWP) can be obtained using echocardiography, but including left atrial (LA) volume (ePAWP-LA) in the estimation may be misleading. We aimed to derive and validate a new estimate without LA volume information (ePAWP-NOLA) and compare its performance to the ASE/EACVI algorithms for diastolic dysfunction.

**Methods and results:**

ePAWP-NOLA was derived and validated in separate datasets of patients who had undergone right heart catheterization and echocardiography. The prognosis was assessed in the validation cohort and the National Echocardiography Database Australia (NEDA) using Cox regression adjusted for age, sex, and left ventricular ejection fraction (LVEF). In the derivation cohort (60 ± 15 years, 40% males, 31% with LVEF < 50%), ePAWP-NOLA was derived from mitral (E), and pulmonary vein systolic (S) and diastolic (D) Doppler velocities (*n* = 134, mean difference ± SD vs. PAWP: 0.0 ± 5.5 mmHg). In the validation cohort (*n* = 116, 51 ± 14 years, 69% males, 89% with LVEF < 50%), PAWP agreed with both ePAWP-NOLA and ePAWP-LA (difference 1.3 ± 6.1, 3.2 ± 6.3 mmHg, respectively). PAWP > 15 mmHg was accurately detected by both ePAWP-NOLA and ePAWP-LA [area under the curve: AUC (95%CI): 0.84 (0.76–0.92), 0.80 (0.72–0.88)]. AUC for the ASE/EACVI algorithm was lower [0.69 (0.61–0.77)]). ePAWP-NOLA and ePAWP-LA correlated with right ventricular afterload and were associated with death or implantation of left ventricular assist device, and with cardiovascular death in NEDA.

**Conclusion:**

ePAWP-NOLA has diagnostic and prognostic performance comparable to ePAWP-LA, and improved diagnostic performance compared to the ASE/EACVI diastolic dysfunction algorithm.

## Graphical Abstract

**Graphical Abstract:**
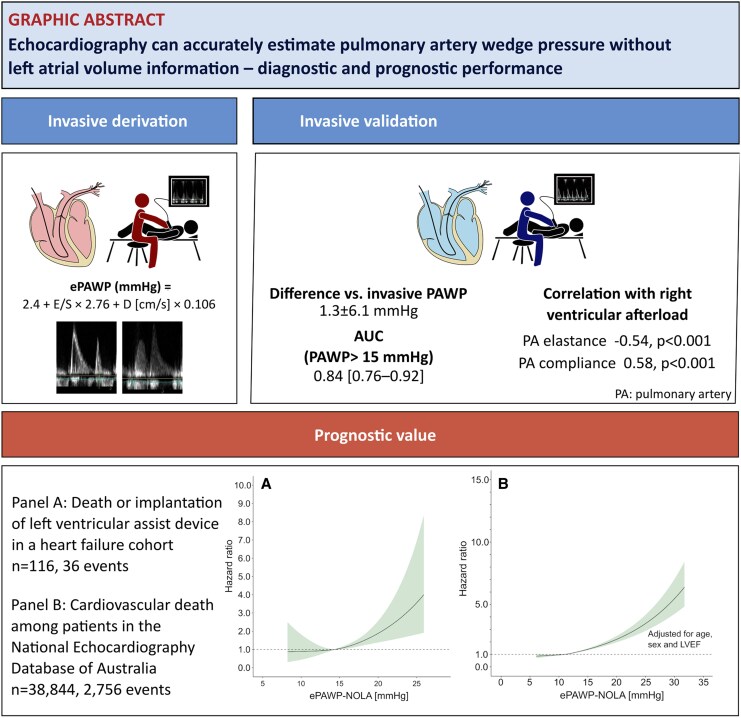

## Background

Quantification of left ventricular filling pressures is important in detection and grading of heart failure (HF). Although invasive techniques like left- or right-heart catheterization (RHC) offer the most precise determination,^1,2^ echocardiography is the preferred bedside method due to its non-invasive nature and widespread accessibility.^3,4^ A noninvasive quantitative estimate of pulmonary artery wedge pressure using echocardiography (ePAWP) based on left atrial (LA) volume, mitral peak early velocity (E), and pulmonary vein systolic velocity (S) was recently described.^5^ This new measure, ePAWP, showed good agreement with invasively measured pulmonary artery wedge pressure (PAWP) in external validation, high accuracy for detection of elevated PAWP, and was associated with cardiovascular death when applied to a large echocardiographic registry.^5^ However, a limitation of the method for estimating ePAWP is its dependency on LA volume. LA remodelling reflects long-term pressure load^6,7^ and although related to PAWP, it may not adapt immediately to changes in PAWP. Also, it is associated with interobserver variability due to differences in the acquisition or focused or non-focused LA views,^8^ and there is a risk of false positive results regarding determining the presence of an elevated PAWP in cases of more prominent LA dilatation or in patients with LA dilatation not caused by increased pressure but arrhythmia (atrial fibrillation) or chronic hyperdynamic circulation (anaemia, liver failure).^9–11^ Therefore, we aimed to derive and validate a quantitative echocardiographic PAWP estimation without LA information (ePAWP-NOLA) in patients with HF symptoms who had undergone echocardiography and RHC, to describe their correlation with measures of right ventricular afterload, and compare its performance to ePAWP with LA information (ePAWP-LA) and the current algorithm for diastolic dysfunction. Furthermore, we sought to assess the prognostic strength of these ePAWP measures both in a selected HF population and in a non-selected, large echocardiographic registry.

## Methods

Four datasets were used in this study, three of them consisting of patients who underwent echocardiography and RHC, the latter based on clinical referral. Two of these datasets, which were used in a previous study to derive and validate ePAWP-LA,^5^ together constituted the derivation population for ePAWP-NOLA. The resulting equation was then applied to a third independent dataset for validation regarding agreement with invasive PAWP, which constituted the primary aim of this study, assessed through bias and precision, and diagnostic accuracy regarding the detection of elevated PAWP. The secondary aim of the validation process was to determine the agreement and correlation of change in PAWP (ΔPAWP) with that of its noninvasive estimates, and the association between ePAWP-NOLA and ePAWP-LA with death or implantation of left ventricular assist device (LVAD). The prognostic value was further evaluated in the large National Echo Database of Australia (NEDA). Ethical approvals were obtained from the Human Research Ethics Committees for each population, respectively.

### Derivation of ePAWP-NOLA

The training set consisted of patients who had undergone RHC at either Karolinska University Hospital, Stockholm, Sweden (*n* = 196) between 2014 and 2018 or Umeå University Hospital, Umeå, Sweden (*n* = 154) between 2010 and 2015. RHC was performed using a Swan-Ganz thermodilution catheter in the right internal jugular vein, a medial cubital vein, or the right femoral vein. PAWP was defined as the mean PAWP and recorded at end-expirium during spontaneous breathing. Patients with at least moderate mitral valve regurgitation, non-sinus rhythms, constrictive pericarditis, missing PAWP measurements, or missing mitral or pulmonary vein velocities were excluded.

**Figure 1:**
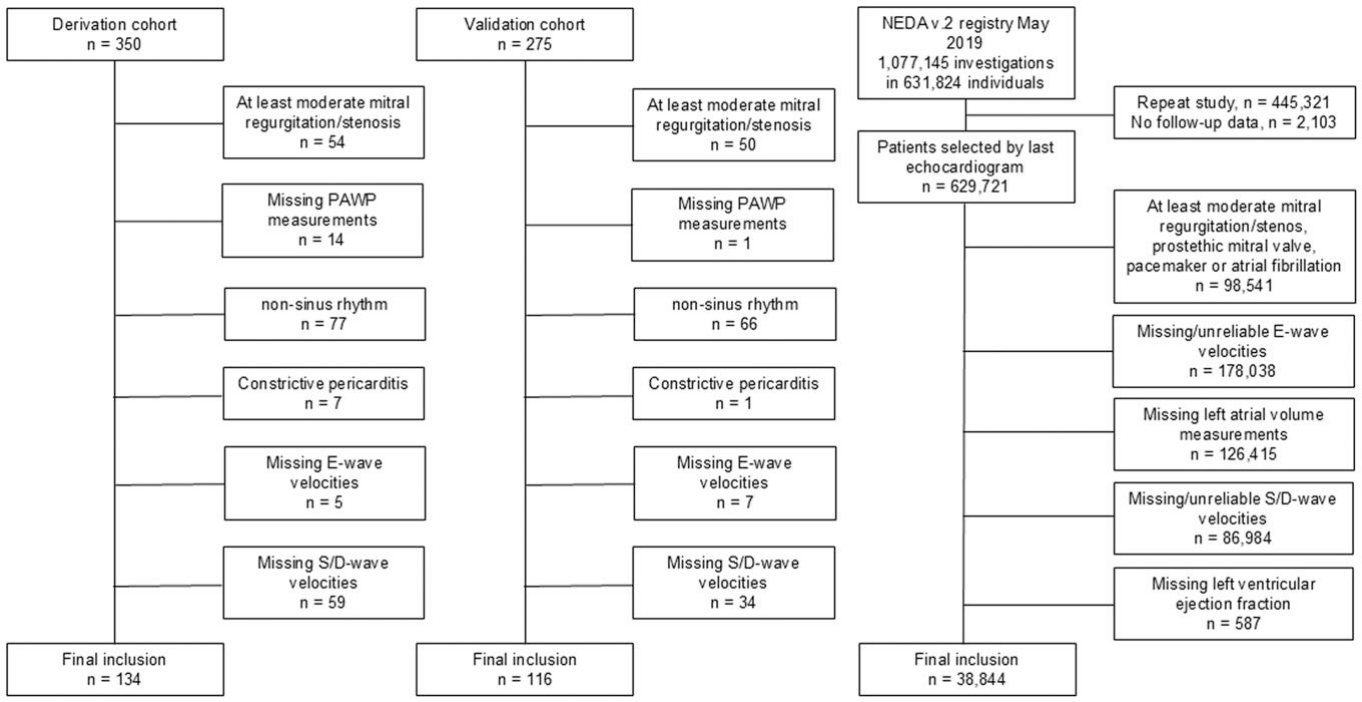
Flowchart of the inclusion and exclusion process for each of the three cohorts (left: derivation cohort; middle: validation cohort; right: NEDA cohort).

The final derivation population consisted of 134 patients for which the main/contributing diagnoses are listed in Supplements, Supplementary data online, *Tables S1* and *S2*. Echocardiographic parameters included in the derivation were mitral E, mitral A, pulmonary vein systolic (S) and diastolic (D) velocities, *e*’, and relevant variable ratios (*E*/*A*, *E*/*e*’, *S*/*D*, *E*/*S*).^5^ Tricuspid regurgitation velocity was deliberately not included, in part because of the high prevalence of absent or non-reliable spectral signals during routine echocardiography,^12^ but in particular due to the association of increased right ventricular systolic pressures and increased pulmonary vascular resistance (PVR) with a broader range of diseases than those causing increased LVFP.^13^ A flowchart of patient exclusions is presented in *Figure 1*.

### Validation of ePAWP measures

The validation population consisted of patients who had undergone clinically indicated RHC and echocardiography in close proximity [median (interquartile range) 1 (1–1) days, 90% within 2 days] of the RHC at Sahlgrenska University Hospital, Gothenburg, Sweden (*n* = 275) between 2009 and 2021. The main indications for catheterization were assessment of haemodynamic status as a part of a work-up for heart transplantation, assessment of pulmonary hypertension or restrictive cardiac physiology, and haemodynamic evaluation in conjunction with an endomyocardial biopsy. Patients with non-sinus rhythms, at least moderate mitral valve regurgitation, constrictive pericarditis, missing PAWP measurements, or missing mitral or pulmonary vein velocities were excluded (*Figure 1*).The final validation population consisted of 116 patients for which the main/contributing diagnoses are listed in Supplements, Supplementary data online, *Table S3*. ePAWP-LA was calculated as previously described,^5^ ePAWP-LA (mmHg) = 0.179 × LAVI (mL/m^2^) + 2.672 × *E* (cm/s)/S (cm/s) + 2.7. In addition, ePAWP estimation based on LAVI and mitral *E* alone, i.e. excluding pulmonary vein velocities, and PAWP estimation based on E/e’ alone were calculated as previously described (ePAWP-E [mmHg]^5^ = 0.230 × LAVI [mL/m^2^] + 0.102 × *E* [cm/s] - 2.7; ePAWP-*E*/*e*’ [mmHg]^14^ = 1.24 × *E*/*e*’ + 1.9). The results for ePAWP-E and ePAWP-E/e’ are presented as supplemental data.

Besides PAWP, invasive measurements of mean pulmonary artery pressure (mPAP), PVR, pulmonary artery compliance (PAC), calculated as stroke volume divided by pulmonary pulse pressure, pulmonary arterial elastance (Ea), calculated as systolic pulmonary artery pressure, divided by stroke volume) were noted for each patient. Through patient records, follow-up was performed until 06 February 2024, to obtain survival status or LVAD implantation, which were used as a composite endpoint.

A subset of these patients (*n* = 52) underwent a repeat RHC and echocardiogram approximately 1 year later. The difference in ePAWP-NOLA and ePAWP-LA, between the first and second examination, is denoted as ΔePAWP-NOLA and ΔePAWP-LA.

### Echocardiography

Comprehensive transthoracic echocardiographic exams were performed in all three populations, using a Vivid E9 system (GE Medical Systems, Horten, Norway). Off-line analyses were done using commercially available image analysis software (EchoPAC, General Electric, Waukesha, Wisconsin, USA). Echocardiograms were analysed by experienced operators blinded to the RHC results. Volumetric and diastolic parameters were obtained and measured according to standard echocardiographic methods.^15^ The ASE/EACVI algorithms^6^ for left ventricular filling pressures, based on *E*/*e*’, LAVI, and systolic pulmonary artery pressure, were applied to all patients.

### Prognostic performance

Prognostic performance of ePAWP measures was also assessed in NEDA, which is a large (>600 000 subjects), observational registry including individual echocardiographic data from participating centres throughout Australia.^16^ Typically, included subjects have been referred for echocardiography by a primary care physician in the investigation of known or suspected heart disease; i.e., these patients did *not* exclusively constitute patients with suspected or known HF. By the structure of the Australian health care system, minimal referral bias applies. Data have then been cross-linked to the Australian National Death Index^17^ to obtain survival status for each subject until the study census date (21 May 2019). In consistency with previous NEDA analyses,^18–20^ causes of death were categorized according to ICD-10, and a primary code within I.00–I.99 was considered as a cardiovascular-related death. This classification has been validated previously.^17^ The NEDA database has been registered in the Australian New Zeeland Clinical Trials Registry (ACTRN12617001387314).

For each eligible subject, only the last echocardiogram (*n* = 629 721) for each eligible subject in NEDA was included. Patients with biological or mechanical mitral valve prosthesis, pacemaker, mitral stenosis, or at least moderate mitral regurgitation, age <18 years, and absent follow-up data were excluded (*n* = 98 541). In addition, cases with missing values on LAVI, E, S, D, or left ventricular ejection fraction (LVEF) and cases with implausible values (pulmonary venous or mitral velocities <0.005 m/s, LAVI <5 mL/m2) were excluded (*n* = 392 024). A flowchart of the exclusion process is presented in *Figure 1*.

ePAWP-NOLA and ePAWP-LA were applied, as well as the American Society of Echocardiography (ASE)/European Association of Cardiovascular Imaging (EACVI) algorithms^21^ for diastolic dysfunction (algorithm 1) and LA pressure (LAP; algorithm 2), based on the presence of reduced/normal LVEF as recommended.^21^ For simplicity, patients with normal diastolic function (ASE/EACVI algorithm 1; LVEF ≥ 50%) and patients with estimated normal LAP (ASE/EACVI algorithm 2; LVEF < 50%) were both classified as having normal diastolic function, those with indeterminate results were classified as having indeterminate diastolic function, and those with abnormal results from either algorithm were classified as having abnormal diastolic function.

In all cohorts, i.e. the derivation and validation cohorts, as well as the NEDA database, cases where missing data required for calculation of ePAWP-LA, ePAWP-NOLA, or classification of diastolic dysfunction according to the EACVI algorithm were excluded. In other words, there were no missing data regarding the calculation of these measures.

### Statistical analysis

Data are presented as mean ± standard deviation (SD) or median (interquartile range) as appropriate. Differences between group medians were performed using the Wilcoxon rank sum test, and differences in proportions using the Chi-square test. Correlations are described using Spearman’s rank correlation coefficient.

ePAWP-NOLA was derived by first applying univariable linear regression to echocardiographic parameters that were presumed to be related to PAWP. In a stepwise selection of variables, multivariable linear regression analysis was then performed through different combinations of variables among those with the highest *R*^2^ in the univariable analysis. Variables were retained in the model only if the adjusted R^2^ increased after addition of a new variable, if a *P* value <0.05 was obtained when comparing models using the likelihood ratio test, and if the Akaike Information Criteria (AIC) was reduced by at least 2.^22–24^ The variance inflation factor (VIF) was determined for the variables included in the final model to detect multicollinearity, with low multi-collinearity pre-specified as VIF <3.

The relation between ePAWP-NOLA, and ePAWP-LA with invasively measured PAWP was described using scatterplots in the derivation and validation cohorts, respectively. Diagnostic performance in the detection of elevated PAWP (>15 mmHg)^2^ was evaluated using receiver operating characteristics (ROC) analysis and described with 95% confidence intervals (95%CI). Comparisons between AUCs were performed using DeLong’s test for correlated ROC curves. Agreement between non-invasive (ePAWP-LA, ePAWP-NOLA) and invasive PAWP estimates was assessed using the Bland–Altman method by plotting the difference between measures against their mean along with 95% limits of agreement (mean ±1.96 × standard deviation of the differences). Agreement between ePAWP measures and invasive PAWP estimates, and AUCs for the detection of elevated PAWP, are also presented for patients with reduced LVEF only ( Supplementary data online, *Table S4*).

In the validation population, the association between ePAWP-NOLA and ePAWP-LA, and the ASE/EACVI diastolic dysfunction grading algorithm respectively, with death or LVAD implantation was evaluated using Cox regression analysis, unadjusted and adjusted for age, sex, LVEF, guideline-directed medical therapy at the time of data collection (beta blocker and renin-angiotensin and aldosterone blockade), and use of loop diuretics. Variables were included as confounders based on subject knowledge and data availability. Proportional hazards were confirmed using Schoenfeld’s residuals. Linearity between ePAWP-NOLA/ePAWP-LA and outcomes was tested using Martingale residuals. A non-linear association was found, and therefore, ePAWP-NOLA or ePAWP-LA were analysed as categorical variables, and as continuous variables using spline regression modelling. The categorization was chosen based on the lowest AIC, between tertiles, quartiles, and cut-offs based on a clinically intuitive cut-off of normal PAWP (≤15 mmHg) and 5 mmHg increments (≤15, > 15–20, 20–25, > 25). The latter yielded the best fit and was used in the final model. A restricted cubic spline regression model was also used to assess the relationship between ePAWP-NOLA and ePAWP-LA and outcomes. The number and placement of knots were determined by selecting the model with the lowest AIC. For the smaller validation cohort, models were tested using either three knots (placed at the 10th, 50th, and 90th percentiles or at the 25th, 50th, and 75th percentiles) or four knots (at the 5th, 35th, 65th, and 95th percentiles). The model with three knots placed at the quartiles of the ePAWP measure yielded the lowest AIC and was therefore selected. In the larger NEDA database, the use of either 3, 4, or 5 knots was tested, and a model using four knots placed at the 5th, 25th, 65th, and 95th percentiles was selected. In the NEDA database, the association between ePAWP-NOLA and ePAWP-LA, and the ASE/EACVI diastolic dysfunction grading algorithm, respectively, and cardiovascular death was evaluated using Cox regression, unadjusted and adjusted for age, sex, and LVEF. In the NEDA database, there are no data on comorbidities or medical therapy. For the purpose of comparison with the categorical values for the ASE/EACVI algorithm, the association with outcomes for ePAWP was also studied after categorizing ePAWP values in a similar way as in the validation cohort (≤15 mmHg, > 15–20 mmHg, > 20–25 mmHg, and >25 mmHg).

A potential interaction effect between ePAWP measures and LVEF on the association with outcomes was evaluated by analysing models with and without an interaction term for LVEF (ePAWP × LVEF). In addition, the prognostic value of ePAWP-NOLA and ePAWP-LA is also presented stratified by LVEF ≥ 50% and <50% (Supplementary data online, *Table S5*).

Model discriminatory performance was assessed using Harrell’s concordance index (C statistics). Hazard ratios (HR) are presented with 95% confidence intervals (95%CI). Comparisons of C statistics between ePAWP measures and the ASE/EACVI algorithm were performed using a z-test for dependent concordance indices. To account for the effect of competing risk of death on the analysis using cardiovascular death as an outcome, competing risks regression as described by Fine and Gray was applied to the multivariable analysis and presented with subhazard ratios (SHR) with 95%CI.^25^ Statistical significance was accepted at the level of *P* < 0.05 (two-sided). Statistical analysis was performed using R version 4.2.1 (R Core Team, Vienna, Austria).

## Results

### Derivation of ePAWP-NOLA

The derivation population consisted of 134 patients (40% males, 60 ± 15 years) with a majority with HF (64.9%) equally distributed between HF with preserved or non-preserved LVEF (34.3% vs. 30.6%). The baseline characteristics of the derivation population are described in *Table 1*. The result of the stepwise linear regression is presented in *Table 2*. Based on this analysis, the best-fit regression equation was ePAWP-NOLA (mmHg) = 2.4 + *E* (cm/s)/*S* (cm/s) × 2.76 + *D* (cm/s) × 0.106. In the derivation cohort, the difference between ePAWP-NOLA and PAWP was 0.0 ± 5.6 mmHg, and the correlation was moderate (*r* = 0.64, *P* < 0.001).

**Table 1:** Baseline characteristics for the derivation and validation cohort, respectively

	Derivation cohort	Validation cohort	*P*
Number of patients, *n*	134	116	
Age, years	62 (51–72)	53 (42–61)	<0.001
Male sex, *n* (%)	53 (39.6)	83 (68.6)	<0.001
NTproBNP, ng/L	561 (259–1961)	1600 (983–3560)	<0.001
HFrEF or HFmrEF, *n* (%)	41 (30.6)	103 (88.8)	<0.001
HFpEF, *n* (%)	46 (34.3)	7 (6.0)	<0.001
Dilated cardiomyopathy, *n* (%)	11 (8.2)	58 (50.0)	<0.001
Hypertrophic cardiomyopathy, *n* (%)	5 (3.7)	5 (4.3)	0.82
Cardiac amyloidosis, *n* (%)	6 (4.5)	4 (3.4)	0.43
Ischaemic heart disease, *n* (%)	18 (13.4)	30 (25.9)	0.01
Pulmonary arterial hypertension, *n* (%)	4 (3.0)	0 (0.0)	NA
BSA, m^2^	1.8 [1.7–2.0]	2.0 [1.8–2.1]	<0.001
Heart rate, min^−1^	70 [61–82]	70 [61–76]	0.30
LV end-diastolic diameter, cm	4.7 [4.2–5.3]	6.3 [5.7–7.0]	<0.001
LVEF, %	60 [45–60]	28 [21–38]	<0.001
LAVI, mL/m^2^	31 [23–45]	43 [35–52]	<0.001
*E*/*A*, unitless	1.2 [0.9–1.9]	2.0 [1.1–3.3]	<0.001
*E*/*e*’, unitless	12 [8–17]	14 [9–18]	0.14
S, cm/s	49 [37–60]	35 [22–47]	<0.001
D, cm/s	51 [39–67]	54 [41–70]	0.58
PAWP, mmHg	13 [9–17]	14 [7–20]	0.82
mPAP, mmHg	24 [19–36]	23 [16–32]	0.03
PVR, Wood units	2.4 [1.4–3.3]	2.0 [1.4–2.6]	0.04

Data are presented as median (interquartile range) or as *n* (%).

BSA: body surface area; HFpEF: heart failure with preserved ejection fraction; HFmrEF: heart failure with mildly reduced ejection fraction; HFrEF: heart failure with reduced ejection fraction; LV: left ventricular; LVEF: left ventricular ejection fraction; LAVI: left atrial volume indexed to body-surface area; PAWP: pulmonary artery wedge pressure.

**Table 2:** Results of the uni- and multivariable regression analyses for prediction of invasive pulmonary artery wedge pressure using echocardiography in the derivation population (***n* = 134)**

	Univariable analysis		Multivariable analysis				
	*R* ^2^	*P*	*Β* coefficient	t	*P*	VIF	Model *R^2^*
*E*/*S*	0.34	<0.001	2.85	7.0	<0.001	1.1	0.41 *P* < 0.001
*S*/*D*	0.23	<0.001					
*E*	0.23	<0.001					
*D*	0.20	<0.001	0.105	4.1	<0.001		
*E*/*A*	0.17	<0.001					
*S*	0.10	<0.001					
*E*/*e*’	0.05	0.02					
Intercept			2.4				

A: mitral A-wave velocity; E: mitral E-wave velocity; e’: mean mitral tissue velocity; D: pulmonary vein diastolic velocity (cm/s); S: pulmonary vein systolic velocity; VIF: variance inflation factor.

Other combinations of variables in the multivariable prediction model did not improve the adjusted *R*^2^ and had higher Akaike Information Criteria.

### Validation of ePAWP measures

Baseline characteristics of the validation population are presented in *Table 1*. Compared to the derivation population, patients in the validation population were younger, had more severe left ventricular systolic dysfunction, higher natriuretic peptide levels, and more often had dilated cardiomyopathy or ischaemic heart disease. PAWP was similar between the derivation and validation cohorts, while mPAP and PVR were slightly higher in the derivation cohort (*Table 1*).

**Figure 2:**
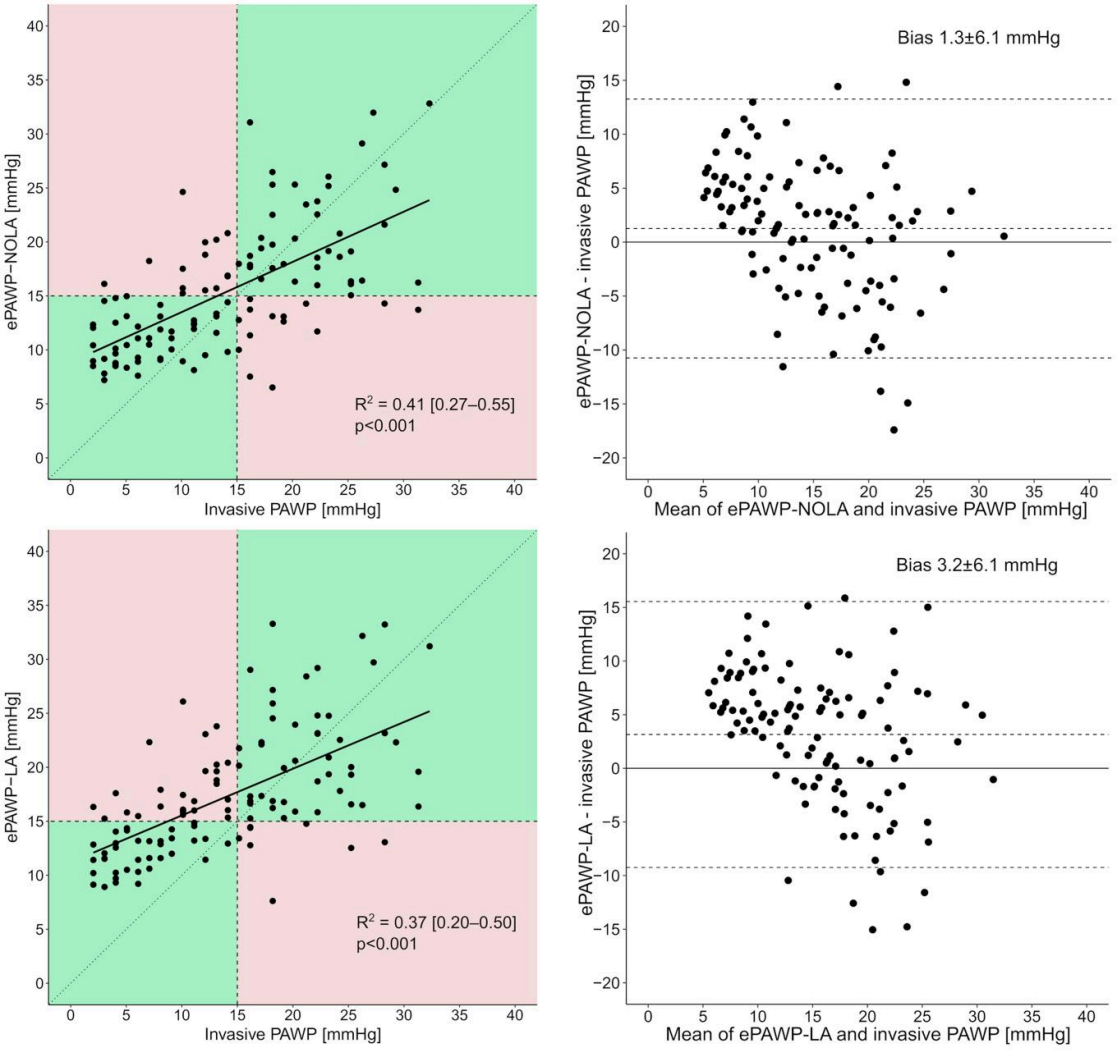
Relation between ePAWP-NOLA (upper panels) and ePAWP-LA (lower panels), with invasively measured pulmonary artery wedge pressure (PAWP) described with scatterplots (left panels) and Bland-Altman plots (right panels) for the validation population (*n* = 116) . In the left panels, upper right and lower left (green) boxes denote correct classifications of either normal or elevated invasively measured PAWP defined as ≤ or >15 mmHg, and upper left and lower right (red) boxes denote incorrect classifications.

**Figure 3:**
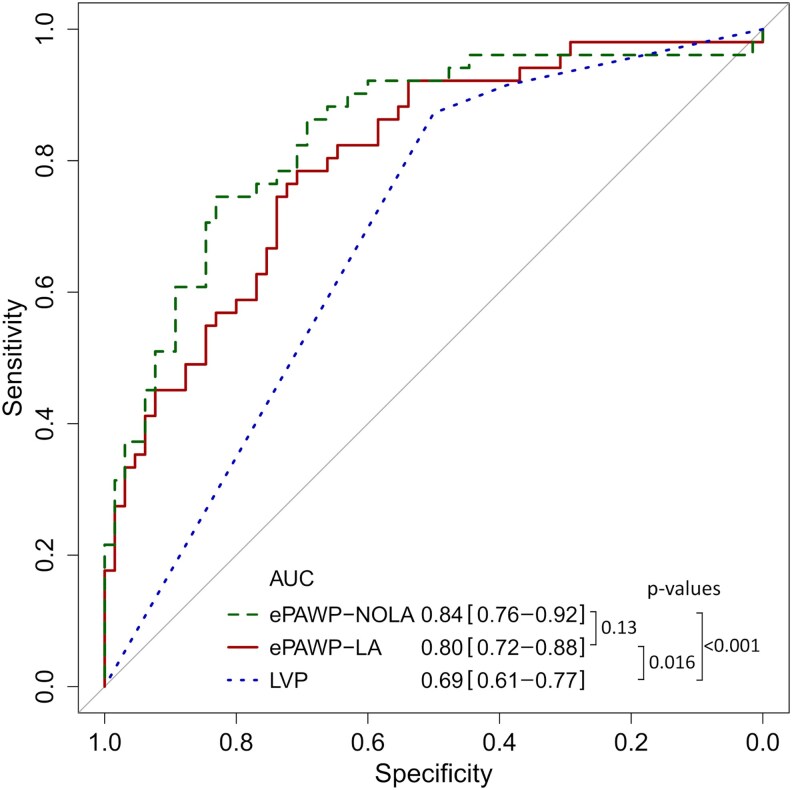
Receiver operating characteristics curve and area under the curve (AUC) with 95% confidence intervals for the detection of elevated pulmonary artery wedge pressure (>15 mmHg) according to right heart catheterization described for ePAWP-NOLA (solid line), ePAWP-LA (dashed line), and left ventricular filling pressures according to the ASE/EACVI algorithm (LVP, dotted line) in the validation population (*n* = 116).^21^

ePAWP-NOLA and ePAWP-LA were applied to 116 patients who had undergone RHC and echocardiography within a median (range) of 1 (0–6) days (90% within 2 days). Among these patients, ePAWP-NOLA had lower bias (1.3 vs. 3.2 mmHg, *P* = 0.02) but similar precision compared to ePAWP-LA (6.1 vs. 6.3 mmHg, *P* = 0.73), using invasively measured PAWP as reference (*Figure 2*). Both ePAWP-NOLA and ePAWP-LA correlated with PAWP (*r* = 0.64, r = 0.61, *P* < 0.001 for both), and with invasive measures pulmonary artery pressure, right ventricular afterload and NT-proBNP (*Table 3*). Both ePAWP-NOLA and ePAWP-LA were superior to the ASE/EACVI algorithm for the detection of elevated LV filling pressures (*Figure 3*). ePAWP-NOLA had the highest specificity and positive likelihood ratio, and ePAWP-LA had the highest sensitivity and lowest negative likelihood ratio, *Table 4*.

**Table 3:** Correlation coefficient (Spearman's ***ρ*) between pulmonary artery wedge pressure (PAWP), ePAWP-NOLA and ePAWP-LA with invasive haemodynamic measures and NT-proBNP in the validation population (*n* = 116)**

	PAWP	mPAP	PVR	Ea	PAC	log NT-proBNP
PAWP	—	0.88*	0.36*	−0.67*	0.84*	0.53*
ePAWP-NOLA	0.66*	0.65*	0.38*	−0.63*	0.67*	0.46*
ePAWP-LA	0.65*	0.63*	0.33*	−0.54*	0.60*	0.47*

^*^ *P* < 0.001.

mPAP: mean pulmonary arterial pressure; PAC: pulmonary artery compliance; PAWP: pulmonary artery wedge pressure; PVR: pulmonary vascular resistance; Ea: pulmonary arterial elastance.

**Table 4:** Diagnostic accuracy of ePAWP-NOLA, ePAWP-LA and the ASE/EACVI algorithm to detect elevated pulmonary artery wedge pressure (>15 mmHg) according to right heart catherization

	Accuracy (%)	Sensitivity (%)	Specificity (%)	PPV (%)	NPV (%)	LR+	ILR-
ePAWP-NOLA >15 mmHg	79	75	83	77	81	4.4	3.3
ePAWP-LA >15 mmHg	71	86	58	62	84	2.1	4.2
ASE/EACVI, LVP↑	67	87	50	61	82	1.7	3.8

LVP, Elevated left ventricular filling pressures according to the ASE/EACVI algorithm^21^; LR+, positive likelihood ration; ILR-, inverse negative likelihood ratio (1/negative likelihood ratio); PPV, positive predictive value; NPV, negative predictive value.

### Detection of change in PAWP

**Figure 4:**
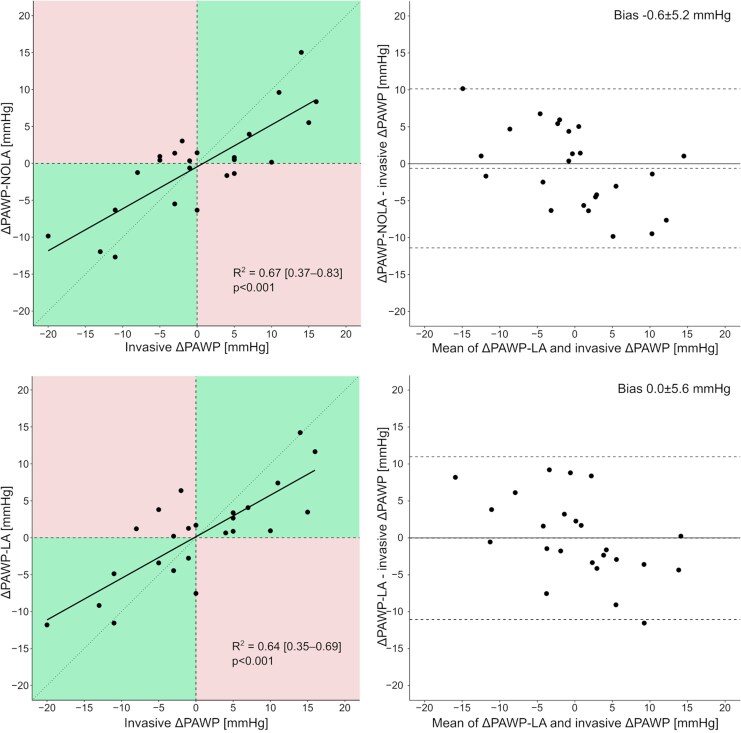
Relation between change (Δ) in ePAWP-NOLA and ΔePAWP-LA and change in invasively measured pulmonary artery wedge pressure (ΔPAWP) described with scatterplots (n = 24). In the left panels, upper right and lower left (green) boxes denote correct classifications of the direction of change of invasively measured PAWP, and upper left and lower right (red) boxes denote incorrect classifications.

Among 52 patients who performed repeated PAWP and echocardiographic measurements, ePAWP-NOLA could be applied to 24 of them. The repeated examination was performed after 302 (125–818) days. ΔPAWP agreed with ΔePAWP-NOLA and ΔePAWP-LA (−0.6 ± 5.2 and 0.0 ± 5.6 mmHg, respectively) and both ΔePAWP-NOLA and ΔePAWP-LA correlated with ΔPAWP [*r* = 0.82 (0.61–0.91), *P* < 0.001 and *r* = 0.80 (0.59–0.91), *P* < 0.001; *Figure 4*].

### Prognostic value of ePAWP measures

**Figure 5:**
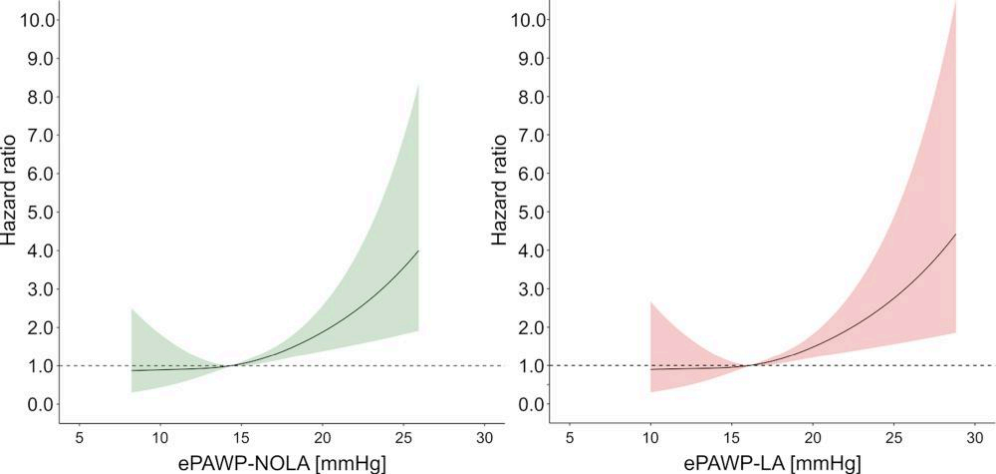
Impact of increasing ePAWP on the risk of death or left ventricular assist implantation for ePAWP-NOLA (left) and ePAWP-LA (right) in the validation population [*n* = 116, follow-up 7 (4–10) years, 36 events]. The hazard ratio was calculated using Cox regression and modelled with natural cubic splines with three knots (25th, 50th, and 75th percentiles) for ePAWP-NOLA (left panel) and ePAWP-LA (right panel).

During a follow-up of 7 (4–10) years, 36 events occurred (31 deaths). In the unadjusted analysis, ePAWP-NOLA and ePAWP-LA >20 mmHg were associated with death or LVAD implantation, and >25 mmHg in the adjusted analysis (*Table 5*). Grading of LV filling pressures according to the ASE/EACVI algorithm was not associated with these outcomes, *Table 5*. The interaction test for LVEF was non-significant for both ePAWP-LA, and for ePAWP-NOLA (*P* = 0.10 and 0.49, respectively). *Figure 5* presents the incremental risk increase with increasing ePAWP-NOLA and ePAWP-LA, respectively. For reference, invasively measured PAWP was associated with death or LVAD implantation [unadjusted HR PAWP >15–20 mmHg: 1.68 [0.70–4.00]; PAWP >20–25 mmHg: 2.62 (1.09–6.33); PAWP > 25 mmHg: 4.74 (1.1.80 12.57), adjusted PAWP >15–20 mmHg: 1.75 (0.71–4.29); PAWP >20–25 mmHg: 2.14 (0.85–5.44); PAWP > 25 mmHg: 6.58 (2.16–20.13)].

**Table 5:** Prognostic value of ePAWP-NOLA, ePAWP-LA and diastolic dysfunction grading according to the ASE/EACVI algorithm presented for both the validation population (***n* = 116, 36 deaths/left ventricular assist implantations) and the NEDA population (*n* = 38,844, 2756 cardiovascular deaths)**

	Validation cohort			NEDA cohort			
	Unadjusted	*C*	Adjusted	Unadjusted	*C*	Adjusted	Adjusted SHR
ePAWP-NOLA							
>15–20 mmHg	1.35 [0.58–3.17]	0.65	1.11 [0.46–2.68]	1.68 [1.43–1.83]	0.53***,^a^	2.02 [1.79–2.29]	1.91 [1.69–2.15]
>20–25 mmHg	3.88 [1.60–9.39]		2.61 [0.98–6.95]	3.78 [2.79–5.09]		2.12 [1.57–2.87]	1.91 [1.42–2.59]
>25 mmHg	5.60 [2.09–15.0]		3.36 [1.15–9.79]	7.55 [4.96–11.5]		3.49 [2.30–5.33]	2.65 [1.79–3.93]
ePAWP-LA							
>15–20 mmHg	1.45 [0.61–3.65]	0.67**	2.18 [0.81–5.87]	3.16 [2.89–3.45]	0.61***	1.84 [1.68–2.01]	1.78 [1.62–1.4]
>20–25 mmHg	3.44 [1.35–8.74]		2.04 [0.68–6.14]	6.53 [5.49–7.77]		2.50 [2.10–2.99]	2.22 [1.88–2.64]
>25 mmHg	4.22 [1.46–12.18]		4.60 [1.45–14.6]	9.78 [7.53–12.7]		3.66 [2.80–4.77]	2.97 [2.24–3.94]
ASE/EACVI							
Indeterminate	0.63 [0.07–5.42]	0.57	0.64 [0.07–5.73]	3.34 [3.06–3.65]	0.65	1.65 [1.51–1.81]	1.61 [1.48–1.76]
LAP↑	2.09 [0.81–5.44]		1.72 [0.63–4.65]	4.87 [4.41–5.37]		1.83 [1.66–2.03]	1.80 [1.63–1.99]

HR: hazard ratio; SHR: subhazard ratio.

The association between ePAWP-NOLA and ePAWP-LA with outcomes is presented for categorical variables with ePAWP ≤15 mmHg as reference. For the ASE/EACVI algorithm, normal diastolic function is the reference.

Comparison of C statistics: ** and *** denote *P* < 0.01 and *P* < 0.001, respectively, compared to ASE/EACVI; ^a^ denotes *P* < 0.001 compared to ePAWP-LA. Validation cohort: ePAWP-NOLA vs. ePAWP-LA *P* = 0.52; ePAWP-NOLA vs. ASE/EACVI *P* = 0.07; ePAWP-LA vs. ASE/EACVI *P* = 0.08; NEDA cohort: ePAWP-NOLA vs. ASE/EACVI *P* < 0.001; ePAWP-NOLA vs. ePAWP-LA *P* < 0.001; ePAWP-LA vs. ASE/EACVI *P* < 0.001.

**Figure 6:**
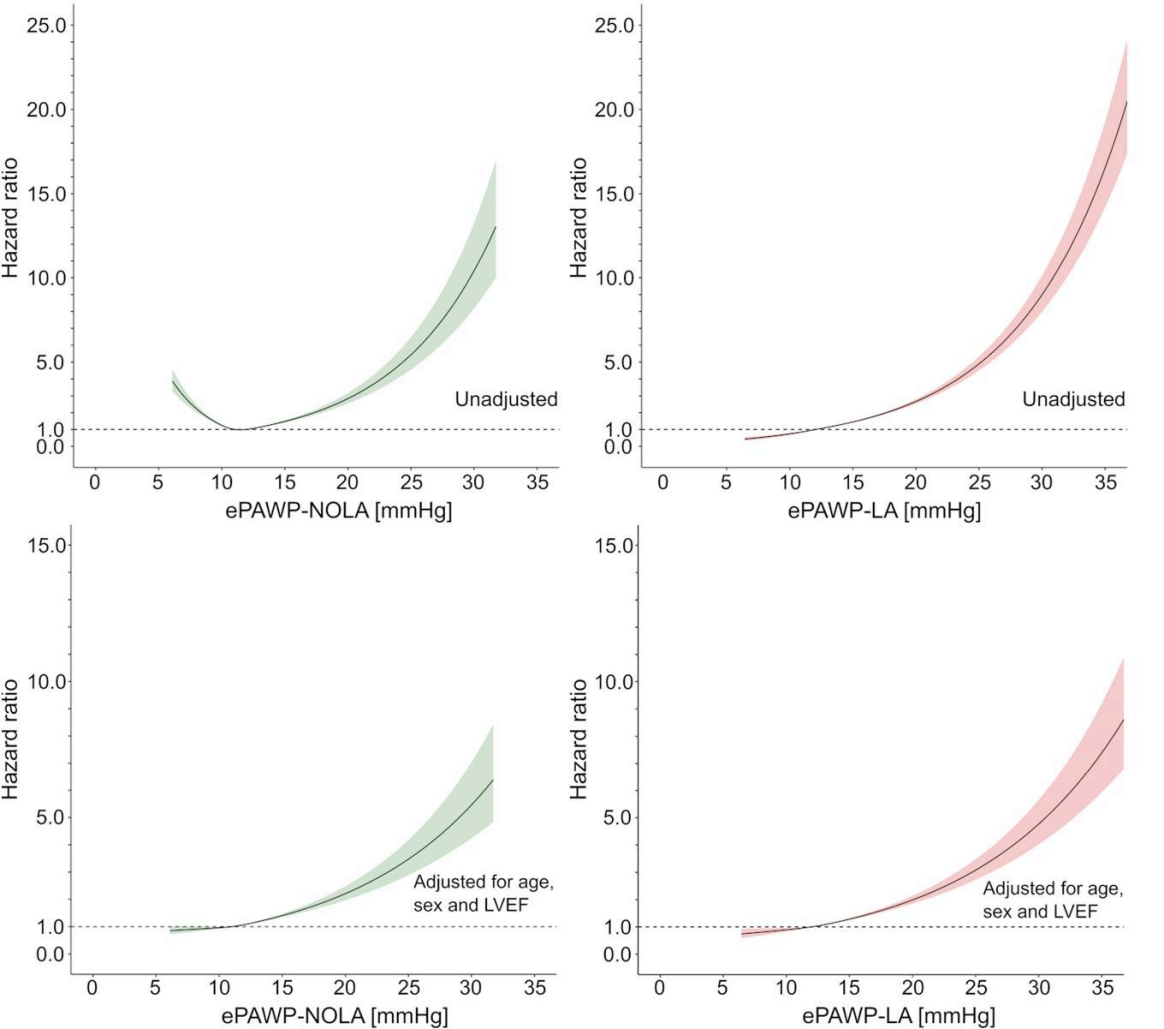
Impact of increasing ePAWP on the risk of cardiovascular death assessed in the National Echocardiography Database of Australia (NEDA), *n* = 38,844, 2756 cardiovascular deaths, unadjusted (upper panels) and adjusted for age, sex, and left ventricular ejection fraction (LVEF) (lower panels). The hazard ratio was calculated using Cox regression and modelled with natural cubic splines with three knots (25th, 50th, and 75th percentiles) for ePAWP-NOLA (left) and ePAWP-LA (right).

In the NEDA population, ePAWP measures were applied to 38 844 patients with a follow-up of 4.8 (2.3–8.0) years, during which 2756 cardiovascular deaths occurred. ePAWP measures were associated with cardiovascular death and similar patterns were observed when accounting for competing risks (*Table 5*). The incremental risk increases with increasing ePAWP values are presented in *Figure 6*. Diastolic dysfunction grading according to the ASE/EACVI algorithm was also associated with cardiovascular death, in the NEDA population.

## Discussion

A quantitative echocardiographic estimation of PAWP can be obtained using pulmonary vein velocities (S and D) and early mitral diastolic velocity (E), thereby eliminating the need to include information on LA size. The regression equation can easily be incorporated into echocardiographic software for automatic and immediate presentation. ePAWP-NOLA derived in patients with echocardiography and RHC showed acceptable accuracy in prediction of PAWP, good ability to detect elevated PAWP, and was associated with incrementally increasing risk of death or LVAD implantation among patients with HF. Change in ePAWP using both ePAWP-NOLA and ePAWP-LA showed a strong correlation with change in invasively measured PAWP. This emphasizes the clinical use of a quantitative PAWP estimate, since algorithmic solutions only can provide information on whether left ventricular filling pressures are elevated or not. When compared to invasively measured PAWP, ePAWP-NOLA showed a numerically lower bias (1.5 mmHg) compared to ePAWP-LA (3.2 mmHg) in PAWP estimation, which contrasts previous reports of low bias for ePAWP-LA vs. PAWP.^5^ However, the current validation cohort consisted of a larger proportion of patients with left heart disease, and HF with reduced ejection fraction (HFrEF) in particular. Relying on LA volume in PAWP estimation would require a linear relation between change in PAWP and change in LA volume, which may not be the case since chronic LA dilatation may remain even in the presence of a reduction in LV filling pressure.^26^ Conversely, from a prognostic perspective, chronic LA dilatation acting as a surrogate for persistently elevated LV filling pressure is strongly associated with a higher mortality. Thus, consideration of both current (ePAWP) and long-term (LAVI) LV filling pressure offers helpful clinical information. However, both ePAWP-NOLA and ePAWP-LA were associated with outcomes, both in the validation cohort and in the larger NEDA database. Significant differences were observed between the derivation group and validation group, for example regarding the prevalence of severe left ventricular systolic dysfunction and aetiology of HF, which strengthens the generalizability of the PAWP estimations across broader HF populations.

Both ePAWP-NOLA and ePAWP-LA had higher diagnostic accuracy than the ASE/EACVI algorithm for the detection of elevated PAWP. For ePAWP-NOLA improvement in specificity was substantial. The superior diagnostic accuracy is in line with previous ePAWP reports,^5^ although the AUC was lower in this study (ePAWP-NOLA 0.84, ePAWP 0.80) compared to the original ePAWP validation populations (0.94).^5^ Compared to the original validation population, the current study consisted of a larger proportion of patients with HFrEF and larger LA volumes. In addition, a quantitative estimate on a continuous scale provides information on the severity of PAWP elevation with greater granularity than the three classes obtained with the ASE/EACVI algorithm. Pulmonary vein velocities are important determinants of both ePAWP-NOLA and ePAWP-LA. The relation between PAWP and pulmonary vein velocities has been described previously.^27–29^ The components of the S wave are affected by both LA relaxation, which in turn is affected by mitral annular plane systolic excursion (MAPSE),^30,31^ right ventricular function, and LA compliance.^28^ Although the S wave is composed of two different waveforms, these are fused to one in the vast majority of cases evaluated by transthoracic echocardiography.^32^ The D-wave velocity, occurring during the LA conduit phase, is affected by the same factors that determine the transmitral pressure gradient as reflected by the mitral E, such as the elastic recoil of the LV and LV stiffness, which will affect PAWP.^33^ The mitral E, and the pulmonary D, is also affected by loading conditions, mitral valve impedance, and blood viscosity.^34^ Given the close relationship between *E* and *D*, it could be presumed that D would carry the same diagnostic information as *E*, and *E*/*S* the same information as *S*/*D*. However, *E*/*S* was more strongly associated with PAWP than *S*/*D*. Moreover, *D* but not *E* provided incremental value to *E*/*S* when determining the regression equation.

Doppler interrogation of pulmonary vein velocities has been described to be difficult and is not currently included in recommendations for routine assessment of LV filling pressures.^21^ Recent studies suggest pulmonary vein velocity acquisitions to be highly feasible,^35^ in particular when included in standard clinical protocols,^5,35^ suggesting that routine exclusion of these measurements is not particularly justified. Jensen, et al. found that antegrade pulmonary vein velocities could be recorded by sonographers with an average of 1 year of experience in 95% of consecutive patients in sinus rhythm referred for transthoracic echocardiography.^35^ High-quality recordings were obtained by placing a 2–3 mm pulsed wave sample volume 1–2 cm into the right upper pulmonary vein using an apical four-chamber view with slight anterior angulation, using colour Doppler to aid identification of the optimal sample volume location. In cases of suboptimal quality, a higher modified apical transducer position, reimaging in the supine position, or subcostal, parasternal, or suprasternal views could increase the success rate.^35^ Pulmonary vein velocity measurements are impacted by intra- and interobserver variability, with coefficients of variation ranging from 5% to 23%,^36^ numbers that are comparable to other echocardiographic measurements.^36,37^ Technical advances are made resulting in improved image quality and more recent studies have described excellent reproducibility for measures of diastolic function, including E-wave velocities.^38^

Both ePAWP-NOLA and ePAWP-LA correlated with invasive measures of increased RV pulsatile afterload (PAC). This strengthens the pathophysiological rationale of including these measures in the assessment of LV filling pressures. An increase in RV afterload is expected in these patients both by backward transmission of increased LA pressure and also as a long-term remodelling effect on the pulmonary vasculature. Both increased resistance and reduced capacitance of the pulmonary vasculature are likely to contribute to reduced S-wave velocities, in particular.^27,32^

We showed a moderate correlation between ePAWP-NOLA and PAWP, and ePAWP-LA and PAWP, respectively. This is in line with the original description of ePAWP,^5^ but contrasts with another recent report.^39^ Prediction of increased PAWP by echocardiography based on the ASE/EACVI algorithm compared to cardiovascular magnetic resonance imaging (CMR) using LA volumes and LV mass has shown a higher accuracy for echocardiography than for CMR.^39^ In a subset of patients in that study, ePAWP could be determined and showed a weak correlation (*r* = 0.34). Bias was not reported, and the number of patients included was small (*n* = 32). Also, no information on the variation of PAWP in that subset was provided.

All ePAWP measures are limited by their precision in reference to invasive PAWP measurements. Of note, even invasive measurements are afflicted by variation at repeated examinations (±2.1 mmHg).^40^ This affects its robustness as a reference standard, e.g. when deriving a quantitative echocardiographic measure, and also puts the observed precision of ePAWP measures in perspective. In that sense, simultaneous measurements of invasive and echocardiographic PAWP would be preferred, but was performed only in a subset of the derivation population (the Umeå cohort). In the Karolinska population, and in the validation, cohort echocardiography was performed in close proximity in time to the right heart catheterization.

The strong accuracy provided by ePAWP-NOLA in reference to PAWP makes this estimation suitable for PAWP estimation in larger studies, allowing for comparisons of PAWP between groups. Also, the strong correlation between change in ePAWP measurements and change in PAWP suggests utility for using ePAWP to assess treatment effects in larger studies.

All ePAWP measurements were associated with an increased risk of death or LVAD implantation, while the ASE/EACVI algorithm was not. In the validation cohort, similarly strong associations were found for invasively measured PAWP. Increasing ePAWP-NOLA and ePAWP-LA were all associated with increasingly higher risk, in line with a previous study of ePAWP.^5^ The HR for ePAWP-NOLA was higher after adjusting for age, sex, and left ventricular ejection fraction. While mitral E and D are higher among younger individuals, S is usually lower. Thus, age is likely to confound the association between ePAWP-NOLA and cardiovascular death with higher ePAWP-NOLA values being more common among the youngest, often healthier individuals. This was confirmed by excluding patients younger than 30 years from the analysis, after which there was an unadjusted association between ePAWP-NOLA and cardiovascular death. In the HF population (the validation population), a strong association with outcomes was found for ePAWP-NOLA even in the unadjusted analysis. This is important, since it suggests that ePAWP-NOLA is robust and provides prognostically relevant information when applied to patients with suspected HF, and ePAWP results must be interpreted in the context of clinical information.

### Limitations

Our findings are limited in part by small sample sizes, the retrospective design, and clinically heterogenous composition of the derivation and validation populations. The adequate performance of ePAWP-NOLA in populations with variable proportions of HFrEF can also be considered a strength allowing for wider application of these estimations.

Another limitation is that no power calculation was made. The datasets were used based on availability. Consequently, external validation in other clinical cohorts is justified.

The evaluation of the prognostic value of ePAWP-NOLA, ePAWP-LA, and diastolic dysfunction in the NEDA database was performed after numerically substantial exclusions, which is a limitation. However, the association with outcomes was found also in the validation cohort, which strengthen the finding of an association between these measures and adverse outcomes.

Also, in the validation cohort, the association between ePAWP measures and death or LVAD implantation was adjusted for age, sex, LVEF, and HF treatment. Residual confounding may be present, particularly in the NEDA cohort, which limits the strengths of the conclusions regarding association with outcomes. Currently, the NEDA database does not include information beyond age and sex—such as comorbidities or medial therapy—restricting the ability to adjust for relevant confounders. However, regarding prognostic value, it is most important to show similar prognostic value between echocardiographic PAWP estimates and invasive PAWP, as this strengthens the similarity between the measures. In clinical practice, the most useful application of the proposed echocardiographic method is the quantitative assessment of LV filling pressures, to determine disease progression or treatment effects. In the context of advanced heart failure, other information is likely to provide greater prognostic value, e.g. peak oxygen consumption or late gadolinium enhancement on cardiovascular magnetic resonance imaging.^41,42^

Nonetheless, these measures need to be validated in prospective, clinical settings in patients with suspected or confirmed HF.

Also, possible differences in echocardiographic acquisition and the absence of a core study laboratory are limitations of our study. In the previous study on ePAWP-LA, interobserver variability was low (0.4 ± 1.0 mmHg) but was assessed only on already acquired images.^5^ Both LA volume and Doppler measurements are dependent on acquisition, and future studies describing the interobserver variability due to differences in acquisition are justified.^8^ However, our results reflect real-world settings. Neither patients with moderate mitral valve lesions, pacemaker, nor atrial fibrillation were included in this study, and ePAWP estimations cannot, therefore, be applied to such patients. Future attempts to derive quantitative PAWP estimations in these patients are merited.

## Conclusion

Compared to ePAWP-LA, ePAWP-NOLA provides comparable diagnostic and prognostic performance, and improved diagnostic performance compared to current diastolic dysfunction guideline algorithms.

## Supplementary Material

qyaf082_Supplementary_Data

## Data Availability

The data underlying this article can be shared upon reasonable request to the corresponding author, except for the data obtained through the National Echocardiography Database of Australia.

## Lead author biography

Thomas Lindow is a consultant at the Department of Clinical Physiology and a cardiology resident at the Department of Medicine, Växjö Central Hospital, Växjö, Sweden. He earned his PhD from Lund University in 2020. In 2021–2022, he completed a postdoctoral fellowship at the University of Sydney under the supervision of Martin Ugander, Professor of Cardiac Imaging. Thomas is affiliated with the Department of Clinical Sciences at Lund University and holds an honorary senior lectureship at the University of Sydney.
