# Supplementary material for: Echocardiography can accurately estimate pulmonary artery wedge pressure without left atrial volume information—diagnostic and prognostic performance
Source: Eur Heart J Imaging Methods Pract. 2025 Jun 13;3(2):qyaf082. doi: 10.1093/ehjimp/qyaf082 (PMC12213055; doi:10.1093/ehjimp/qyaf082)
Supplement: qyaf082_Supplementary_Data [file qyaf082_supplementary_data.docx]

**SUPPLEMENTS**

| **Table S1. Principal and/or contributing diagnoses after right heart catheterization in the Karolinska cohort (part of the derivation cohort)** | |
| --- | --- |
| Heart failure with preserved ejection fraction | 17 (18.9) |
| Heart failure with reduced/mildly reduced ejection fraction | 10 (11.1) |
| Ischemic heart disease | 9 (10) |
| Pre-/post heart transplantation | 6 (6.7) |
| Restrictive cardiomyopathy | 5 (5.6) |
| Right ventricular failure | 5 (5.6) |
| Pulmonary fibrosis | 5 (5.6) |
| Normal | 4 (4.4) |
| Aortic stenosis | 4 (4.4) |
| Chronic thromboembolic pulmonary hypertension | 4 (4.4) |
| Systemic sclerosis | 4 (4.4) |
| Dilated cardiomyopathy | 3 (3.3) |
| Cardiac amyloid | 3 (3.3) |
| Hypertrophic cardiomyopathy | 3 (3.3) |
| Myocarditis | 2 (2.2) |
| Pulmonary hypertension | 2 (2.2) |
| Chronic obstructive pulmonary disease | 2 (2.2) |
| Arythmogenic cardiomyopathy | 1 (1.1) |
| Cirrhosis | 1 (1.1) |
| Idiopathic pulmonary arterial hypertension | 1 (1.1) |
| Respiratory insufficiency | 1 (1.1) |

| **Table S2. Principal and/or contributing diagnoses after right heart catheterization in the Umeå cohort (part of the derivation cohort)** | |
| --- | --- |
| Heart failure with reduced/mildly reduced ejection fraction | 11 (20.8) |
| Systemic sclerosis | 6 (11.3) |
| Normal | 5 (9.4) |
| Heart failure with preserved ejection fraction | 5 (9.4) |
| Associated pulmonary arterial hypertension | 5 (9.4) |
| Chronic thromboembolic pulmonary hypertension | 5 (9.4) |
| Systemic lupus erythematosus /Mixed connective tissue disease | 4 (7.5) |
| Idiopathic pulmonary arterial hypertension | 3 (5.7) |
| Ischemic heart disease | 2 (3.8) |
| Chronic obstructive pulmonary disease | 2 (3.8) |
| Hypertrophic cardiomyopathy | 1 (1.9) |
| Aortic stenosis | 1 (1.9) |
| Ventricular septum defect | 1 (1.9) |
| Cardiac amyloid | 1 (1.9) |

| **Table S3. Principal and/or contributing diagnoses after right heart catheterization in the validation cohort** | |
| --- | --- |
| Myocarditis | 4 (3.4) |
| Amyloidosis | 4 (3.4) |
| Aortic regurgitation | 3 (2.6) |
| Dilated cardiomyopathy | 57 (49.1) |
| Peripartal cardiomyopathy | 1 (0.9) |
| Hypertrophic cardiomyopathy | 5 (4.3) |
| HFpEF | 1 (0.9) |
| Hypertension | 1 (0.9) |
| Ischemic heart disease | 30 (25.9) |
| Unspecified | 4 (3.4) |
| Myositis | 2 (1.7) |
| Sarcoidosis | 2 (1.7) |
| Restrictive cardiomyopathy | 2 (1.7) |

**Regression equation for estimation of pulmonary arterial wedge pressure (PAWP) using only left atrial volume indexed to body surface area (LAVi) and mitral early peak velocity (E) or E/e’**

*ePAWP_E =_ 0.230 × LAVi + 10.177 × mitral E - 2.7,* in which ePAWP is given in mmHg, LAVi in ml/m^2^ and mitral E in m/s

*ePAWP-E/e’ [mmHg]^12^ = 1.24 × E/e’ + 1.9*

*Comparison of PAWP estimation ePAWP-E and ePAWP-E/e’*

ePAWP-NOLA, ePAWP-LA, ePAWP-E and ePAWP-E/e’ could be applied to the same 79 patients in the validation cohort. In this subset, ePAWP-E/e’ had larger bias and worse precision (6.5±11 mmHg) compared to ePAPW-NOLA (1.3±6.1 mmHg), ePAWP-LA (3.2±6.4 mmHg) and ePAWP-E (0.1±6.7).

| **Table S4.** Agreement of ePAWP-NOLA and ePAWP-LA with invasively measured pulmonary artery wedge pressure (PAWP) among patients with reduced left ventricular ejection fraction (<50%) in the validation cohort (n=103) | | | |
| --- | --- | --- | --- |
|  | Agreement (mmHg) | AUC for detection PAWP >15 mmHg |  |
| ePAWP-NOLA | 1.1±6.2 | 0.83 [0.75–0.91] | p=0.80 |
| ePAWP-LA | 3.2±6.3 | 0.80 [0.72–0.89] |  |

| **Table S5.** Prognostic value of ePAWP-NOLA, ePAWP-LA and diastolic dysfunction grading according to the ASE/EACVI algorithm stratified by left ventricular ejection fraction (LVEF) in the NEDA population | | | | | | | |
| --- | --- | --- | --- | --- | --- | --- | --- |
|  | LVEF>50%  (n=35,488, 2,131 cardiovascular deaths) | | | | LVEF<50%  (n=3,356, 625 cardiovascular deaths) | | |
|  | HR, unadjusted | C | HR, adjusted | HR, unadjusted | | C | HR, adjusted |
| *ePAWP-NOLA* |  |  |  |  | |  |  |
| >15-20 mmHg | 1.45 [1.25–1.68] | 0.52 | 2.05 [1.77–2.37] | 1.93 [1.54–2.43] | | 0.56 | 2.02 [1.61–2.53] |
| >20-25 mmHg | 3.49 [2.33–5.22] |  | 2.22 [1.48–3.32] | 1.93 [1.21–3.05] | |  | 1.91 [1.21–3.02] |
| >25 mmHg | 7.41 [4.45–12.3] |  | 3.76 [2.26–6.26] | 4.55 [2.16–9.61] | |  | 3.73 [1.77–7.87] |
| *ePAWP-LA* |  |  |  |  | |  |  |
| >15-20 mmHg | 1.45 [0.61–3.65] | 0.67 | 1.89 [1.70–2.11] | 1.92 [1.60–2.29] | | 0.61 | 1.58 [1.41–2.01] |
| >20-25 mmHg | 3.44 [1.35–8.74] |  | 2.84 [2.28–3.52] | 2.69 [2.00–3.63] | |  | 2.08 [1.54–2.82] |
| >25 mmHg | 4.22 [1.46–12.18] |  | 3.88 [2.73–5.52] | 5.74 3.85–8.56] | |  | 4.24 [2.84–6.34] |
| *ASE/EACVI* |  |  |  |  | |  |  |
| Indeterminate | 3.63 [3.01–3.69] | 0.64 | 1.57 [1.42–1.75] | 1.61 [1.34–1.94] | | 0.57 | 1.68 [1.40–2.02] |
| LAP↑ | 5.31 [4.74–5.95] |  | 1.89 [1.66–2.13] | 1.57 [1.28–1.91] | |  | 1.50 [1.23–1.83] |
| **Abbreviations**: HR: hazard ratio; LAP: Left atrial pressure; LVEF: left ventricular ejection fraction  The association between ePAWP-NOLA and ePAWP-LA with outcomes are presented for categorical variables with ePAWP ≤15 mmHg as reference. For the ASE/EACVI algorithm, normal diastolic function is the reference. | | | | | | | |

**
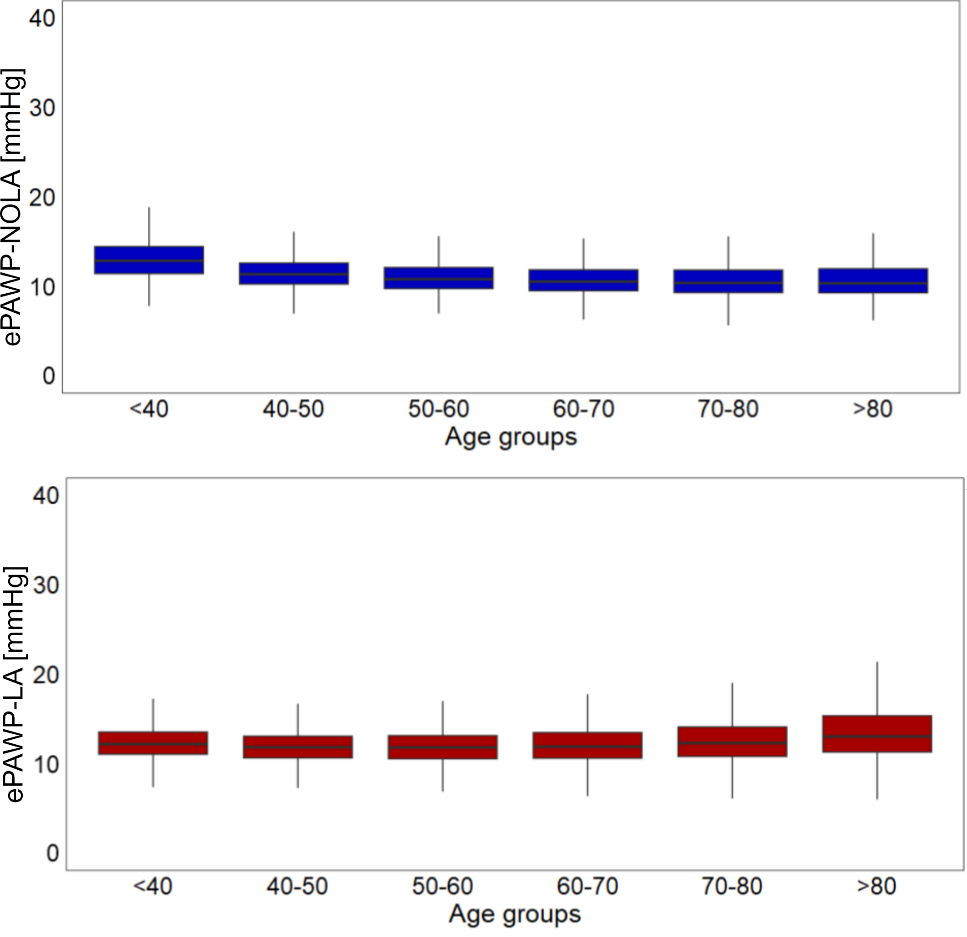
**

**Figure S1.** Distribution ePAWP-NOLA (upper panel) and ePAWP-LA (lower panel) stratified by age among patients in the NEDA population. Notably, ePAWP-NOLA are highest for younger individuals.
